# Patterns and Associations of Essential Trace Elements (Cu, Fe and Zn) in Saudi Adults with Varying Levels of Glycemia

**DOI:** 10.3390/metabo11050297

**Published:** 2021-05-06

**Authors:** Sobhy Yakout, Fatimah Faqeeh, Omar Al-Attas, Syed D. Hussain, Nasser M. Al-Daghri

**Affiliations:** Chair for Biomarkers of Chronic Diseases, College of Science, King Saud University, Riyadh 11451, Saudi Arabia; sobhy.yakout@gmail.com (S.Y.); fatimah.faqeeh@hotmail.com (F.F.); omrattas@ksu.edu.sa (O.A.-A.); danishhussain121@gmail.com (S.D.H.)

**Keywords:** type 2 diabetes, prediabetes, trace minerals, Arabs

## Abstract

The homeostasis of trace elements were observed to contribute to certain diabetic outcomes. This cross-sectional study determined the differences and associations between serum levels of copper (Cu), iron (Fe) and zinc (Zn) in Saudi patients with and without type 2 diabetes mellitus (T2DM) as well as those with prediabetes. Anthropometrics were measured, and fasting blood samples were collected from 119 patients with T2DM (aged 41–64 years), 95 non-T2DM (aged 27–55 years) and 80 with prediabetes (aged 35–57 years). Circulating trace minerals were determined using an inductively coupled plasma–mass spectrometer. Serum levels of Cu and Fe were significantly lower in T2DM than non-T2DM (adjusted *p*-values < 0.001). There was no difference in the Zn levels of the T2DM and non-T2DM groups. The serum Cu levels were significantly lower in the prediabetes group than the non-T2DM group (*p* < 0.05). The serum levels of Cu, Fe and Zn were inversely associated with circulating glucose in the T2DM and prediabetes subjects (*p*-values < 0.001). In conclusion, the differences in circulating trace elements were observed in Saudi subjects with varying glycemic statuses, suggesting an inverse association between T2DM progression and the decreasing serum Cu, Fe and Zn levels. Intervention trials are warranted to determine whether early correction of trace mineral deficiencies is beneficial in populations at higher risk for T2DM.

## 1. Introduction

Diabetes mellitus (DM) is still growing in prevalence worldwide and is becoming a serious threat to human health [1]. The estimated prevalence of DM in adults aged 20–70 years worldwide was 8.3% in 2013 and is expected to increase to 10.1% by 2035 [2]. In recent years, age-adjusted epidemiologic studies have estimated that the predominance of type-2 diabetes mellitus (T2DM) in the Kingdom of Saudi Arabia (KSA) was 31.6% in 2011 alone [3]. This high prevalence has led to concerted efforts within the local research community in determining the genetic factors unique to the population [4,5] as well as promising adjuvant interventions for the Saudi Arabian T2DM population independent of established standard care [6,7,8]. Despite the accomplishments in acquiring new knowledge about T2DM that are specific to the Arabian ethnicity, the multifactorial nature of T2DM opens more avenues for investigation, and certain factors such as the influence of micronutrients and trace elements remain under studied.

Trace minerals are known to play a key role in the action or secretion of insulin [9]. They can be divided into essential trace elements that are critical for life sustainability and processes, and nonessential trace elements [10]. Trace elements are micronutrients required by the body to perform its normal functions. Although they have several vital roles in human metabolism, chronically excessive or reduced concentrations of these elements can be toxic for the body’s health and may lead to several disorders such as T2DM [11].

Several essential trace elements contribute to the development or prevention of T2DM. Copper (Cu) is required for several biological roles and is one of the metalloenzymes that play a significant role in the catalytic action of superoxide dismutase (SOD), which participates in protecting cells from superoxide radicals [12]. Cu is also needed to activate cytochrome oxidase in the mitochondria, which is engaged in the electron transport chain. A Cu deficiency inhibits cytochrome oxidase activity and may cause distortion of the mitochondria in metabolically active tissues such as pancreatic acinar cells and hepatocytes [13]. Cu is also needed in the formation of red blood cells as well as in the maintenance of the circulatory, nervous, immune and skeletal systems [12].

Iron (Fe) is important in the synthesis of hemoglobin. Furthermore, it is necessary in the production of elastin, collagen, ascorbic acid synthesis and Zn [13]. It has been suggested that Fe has a role in the development of T2DM through multiple mechanisms, including in the modulation of insulin sensitivity and resistance, and causes hepatic dysfunction [14]. Furthermore, it has been established that decreased levels of Fe increases the glycation of HbA1C [15], which can lead to false high values of HbA1c even in people without DM [16].

Zinc (Zn) is an essential part of many enzymes and is a cofactor in many catalytic activities [13,17]. It serves important roles in the maintenance of several tissue functions such as the synthesis, release and storage of insulin [17]. A loss of enzymatic activity can occur if Zn is removed from the catalytic site of an enzyme [13]. Zn plays an important role in glucose metabolism, which is found to enhance insulin sensitivity [14]. Abnormal levels of Zn may contribute to the pathogenesis and some complications of T2DM [18]. Zn is a key controller of glucose metabolism based on its link to several gluconeogenic enzymes such as glucose 6-phosphatase and phosphoenolpyruvate carboxykinase [19]. Studies have observed an association between the status of trace elements such as Zn and the action, production and release of insulin [20].

The present study aims to determine the patterns of essential trace elements such as Cu, Fe and Zn in Saudi adult subjects with varying levels of glycemia and to determine the associations between these trace elements among the T2DM and prediabetes subjects. 

## 2. Results

The studied subjects’ characteristics are shown in Table 1. Among the 294 subjects included, 40% have T2DM, 35% have non-T2DM and the remaining 25% have prediabetes. Table 1 demonstrates that the mean systolic and diastolic blood pressures were significantly higher in T2DM patients and the prediabetes group as compared to healthy controls. The mean age of the prediabetes group was 46.7 ± 10.9 years, and the T2DM group had a mean age of 52.2 ± 11.2 years. The majority of the subjects in the prediabetes and T2DM groups were overweight (BMI 25.0–29.9 kg/m^2^), and a few of the subjects were obese (BMI ≥ 30 kg/m^2^). 

Table 2 shows the differences in circulating trace elements of subjects. The mean serum Cu levels of the T2DM and the prediabetes groups were significantly lower than their non-T2DM counterparts, even after adjusting for age, BMI and sex. The mean serum Fe levels of the T2DM group were significantly lower than both the prediabetes and non-T2DM groups. The mean serum Zn levels of the prediabetes group was significantly higher compared to T2DM and non-T2DM groups (Table 2).

### Correlations between Serum Glucose and Trace Elements in T2DM and Prediabetes Subjects

**Figure 1:**
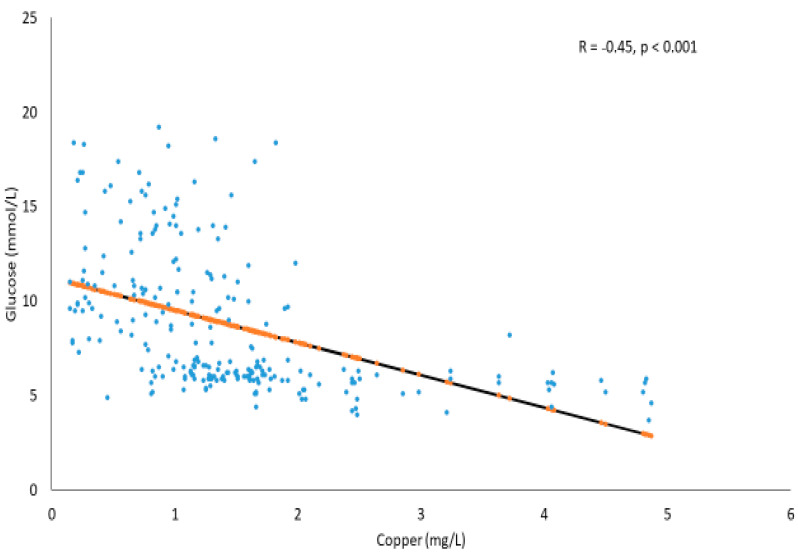
Correlation between glucose (mmol/L) and Cu (mg/L) in T2DM and prediabetes subjects.

Figure 1 shows the significant inverse association between glucose (mmol/L) and Cu (mg/L) in the T2DM and prediabetes subjects (R = −0.45, *p* < 0.001). The trend line also suggests that, as the Cu levels increase, glucose concentrations tend to decrease moderately. 

**Figure 2:**
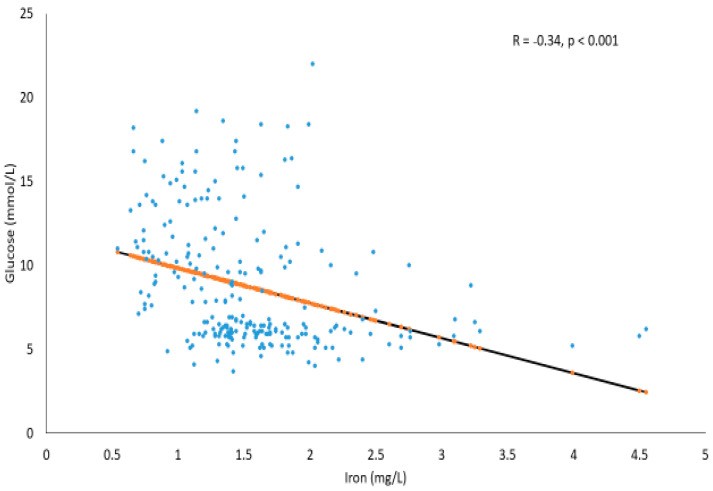
Correlation between glucose (mmol/L) and Fe (mg/L) in T2DM and prediabetes subjects.

Figure 2 shows the significant inverse association between glucose (mmol/L) and Fe (mg/L) in the T2DM and prediabetes subjects (R = −0.34, *p* < 0.001). The trend line also suggests that, as Fe increases, glucose concentration tends to decrease modestly.

**Figure 3:**
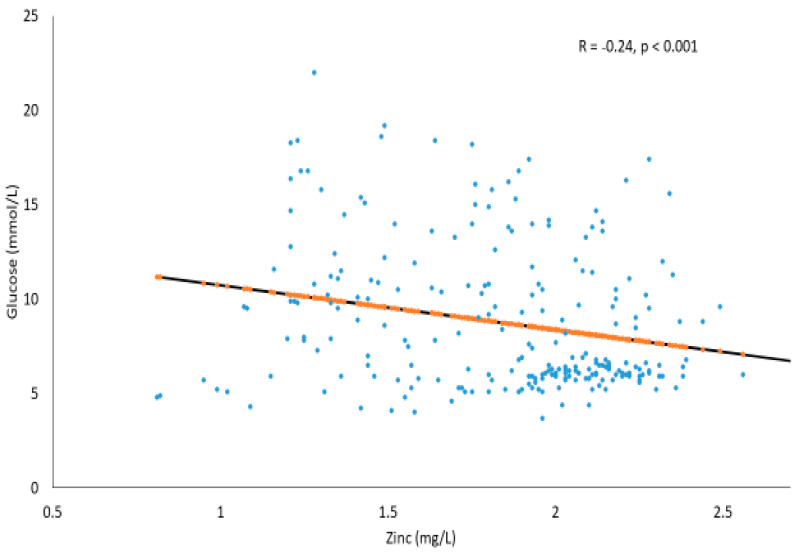
Correlation between glucose (mmol/L) and Zn (mg/L) in T2DM and prediabetes subjects.

Figure 3 shows the significant inverse association scatter plot between glucose (mmol/L) and zinc (mg/L) in the T2DM and prediabetes subjects (R = −0.24, *p* < 0.001). The trend line also suggests that, as the Zn levels increase, glucose concentration tends to decrease mildly.

## 3. Discussion

Clearly, the Cu levels in the T2DM group were significantly lower than both the non-T2DM and the prediabetes groups. In addition, Cu levels in the prediabetes group was lower than the non-T2DM group. Several studies showed similar results for lower Cu concentrations in the T2DM population [12,20,21,22]. Furthermore, when compared with healthy control subjects, Cu was considered deficient in their T2DM counterparts [23,24]. Low plasma concentrations of Cu can lead to DM as a result of oxidative stress [24]. The impairment of glucose tolerance can be secondary to a deficiency of Cu [25], which was used as an indicator of impaired glucose tolerance in an earlier study [21]. The deficiency of Cu produces swelling following the disruption of the mitochondria of metabolically active tissues, such as pancreatic cells and hepatocytes [26]. The proper dietary intake of Cu may inhibit T2DM development due to its antioxidant properties, while a low dietary intake of Cu is associated with a higher risk of T2DM [27]. 

Fe levels in the T2DM group was significantly lower than other groups. Several studies showed a significantly lower Fe concentration in the T2DM patients when compared to the healthy controls [15,16,24,28]. Fe supplementation is suggested to be a target therapy for patients at risk of T2DM [24]. A study by Aljohani et al., 2018, showed that a high incidence of anemia is more likely to occur in patients with poorly regulated diabetes and in T2DM patients with renal insufficiency [28]. Naqash et al. reported that lower levels of serum Fe have been associated with higher levels of HbA1c [15]. Some authors focused on studying HbA1c levels and anemias, such as iron deficiency anemia (IDA) [29]: a chronic disorder attributed to a reduction in the production of red blood cells that may cause an increase in HbA1c [30]. A significant improvement in the levels of HbA1c was reported in various studies after treatment with iron supplements [31,32]. The association between the HbA1c levels and IDA has been established and shows that there are factors other than glucose that can affect HbA1c, and this should be taken into consideration [32]. Finally, the outcomes are conflicting regarding the link between iron and diabetes [24]. 

The Zn levels in the prediabetes group were significantly higher than that of the non-T2DM and the T2DM group. No significant difference was found in Zn levels of the T2DM and control groups. There are studies that agree with our results that demonstrated no significant difference between the mean Zn levels of the T2DM subjects and the healthy control subjects [25,33]. Moreover, another study demonstrated that the levels of Zn are not altered in subjects with T2DM [34]. For instance, low levels of Zn in the plasma of prediabetes and DM subjects have been observed in previous studies [35]. In contrast, a study by Vashum et al., 2014, reported that the prediabetes group has high levels of Zn, which is associated with increased insulin sensitivity and B-cell function [36]. The association between Zn and insulin resistance can be partially explained by Zn ions’ inhibition of protein tyrosine phosphatase 1B, a crucial regulator of the active phosphorylation form of the insulin receptor [35]. In addition, Zn, being a cofactor of antioxidant enzyme superoxide dismutase, can cause oxidative stress that further stimulates the stress pathways that cause insulin resistance [35]. It is suggested that Zn deficiency could be associated with a defect in insulin secretion due to its insulin-like effects via inhibition of the glycogen-regulating enzyme GSK3, activation of post-receptor proteins Akt and PI3 kinase, and the reduction of cytokines IL-1b and NFĸB [35]. Furthermore, the reduction in the blood concentrations of Zn from these patients was linked with an increased urinary excretion of Zn [37,38]. Some studies demonstrated that Zn supplementation may provide an important protection against diabetes as complications of diabetes were highly linked with Zn deficiency [39]. This relationship, however, is not clear [40].

In T2DM, the imbalances between specific metals might have a role in upsetting normal glucose and insulin metabolism, and variations in the status of trace minerals can also elevate the oxidative stress that might cause insulin resistance and diabetic complications. The outcomes showed that T2DM status is associated with both low concentrations of Cu and Fe. In this context, the results suggest that a proper dietary intake of Cu may be beneficial given its antioxidant properties. The link between Fe deficiency and diabetes needs further exploration.

The authors acknowledge the following limitations: the cross-sectional design limits causality and the hematological indices were not assessed to rule out anemia, especially since circulating Fe was assessed.

## 4. Materials and Methods

The sample size calculation was carried out using an effect size of 0.2 at 80% power and a significance level of 0.05 with three groups including T2DM, prediabetes and non-T2DM. The total required sample size was 256. This cross-sectional study included 294 subjects (aged 25–65 years) whose clinical information and blood samples were retrieved from an existing database of the Chair for Biomarkers of Chronic Diseases (CBCD) in King Saud University, Riyadh, KSA. 

Our study consisted of three groups: prediabetes (group 1), T2DM (group 2) and non-T2DM controls (group 3). Patient cases were categorized as diabetes and prediabetes according to the World Health Organization criteria as implemented in previous studies [3]. Fasting blood glucose levels above 7.0 mmol/L were considered T2DM while those between 6.1 and 7.0 mmol/L were considered prediabetic.

Ethical approval was obtained from the Ethics Committee of the College of Science Research Center, King Saud University, Riyadh, Saudi Arabia (approval No. 8/25/454239). Written informed consent was obtained from all subjects involved in the study. All participants completed a questionnaire on demographic information, general health status and past medical history. A physical examination was carried out by the attending physician who ensured the exclusion of subjects with conditions that required immediate medical attention such as cardiac, kidney or liver disease; psychiatric conditions; and use of medications. The anthropometrics that were taken included BMI (kg/m^2^) and blood pressure (mmHg).

### 4.1. Trace Element Determination

The trace metal analytical determination was carried out using an ICP-MS (Inductively Coupled Plasma-Mass Spectrometer): NexION 300 D (Perkin Elmer, Waltham, MA, USA). An ICP-MS consists of a high-temperature ICP (Inductively Coupled Plasma) source and a mass spectrometer. First, the Inductively Coupled Plasma source transfers the atoms of the elements in the sample to ions. Then, these ions are separated and observed by the mass spectrometer. Table 3 highlights the operating conditions of the instruments used in this study.

**Figure 4:**
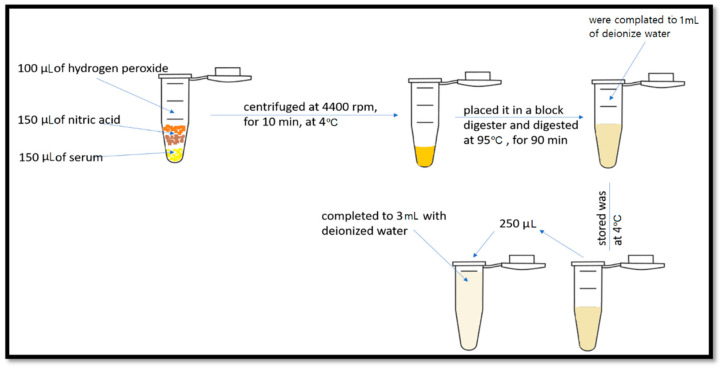
Preparation steps of the sample for the high-resolution sector field ICP-MS.

Several steps were needed to prepare the sample for high-resolution sector field ICP-MS, as shown in Figure 4.

### 4.2. Statistical Analysis

The data were analyzed using SPSS version 21. Continuous data were presented as a mean and standard deviations for normal variables and as a median (first quartile–third quartile) for non-normal variables. Furthermore, categorical data were presented as frequencies and percentages (%). ANOVA was used to determine the significant mean difference between groups for normal variables, while a Kruskal–Wallis analysis was used to test the significant median difference between groups for non-normal variables. A chi-squared test was used to determine the differences in proportions between the categorical variables. The Pearson correlation coefficient was used to determine the correlation between select variables. All non-normal variables were log-transformed prior to parametric testing. *p* < 0.05 was considered significant.

## 5. Conclusions and Recommendations

In summary, the serum concentrations of Cu and Fe in T2DM patients were significantly lower than those in the healthy control group and those with prediabetes. Significant inverse correlations between the serum concentrations of these trace elements and glucose were apparent in both the T2DM and prediabetes groups. These observations suggest that the patterns of circulating trace elements are modified depending on the glycemic status of the subject and that a correction of trace element deficiencies might prove beneficial among individuals at high risk For T2DM. Further prospective studies are needed to confirm this. In conclusion, the serum profile of some trace elements may be used by healthcare providers to monitor deficiencies and to identify individuals at high risk for T2DM.

## Figures and Tables

**Table 1:** Demographic and clinical characteristics of subjects according to T2DM status.

Parameters	Non-T2DM	Prediabetes	T2DM	*p*-Value	*p*-Value *
N	95	80	119	--	--
Age (Years)	40.9 ± 13.7	46.7 ± 10.9	52.2 ± 11.2 ^A,B^	0.000	--
Female/Male	68/27	49/31	57/62	0.002	--
BMI (kg/m^2^)	29.9 ± 6.0	32.6 ± 7.6	31.8 ± 6.0	0.017	--
WHR	0.9 ± 0.1	0.9 ± 0.1	1.0 ± 0.1	0.003	0.13
Systolic BP (mmHg)	112.3 ± 10.3	124.3 ± 14.1 ^A^	128.5 ± 15.1 ^A^	<0.001	<0.001
Diastolic BP (mmHg)	74.1 ± 7.0	78.5 ± 8.0	79.6 ± 9.0	0.004	0.06

Note: data are presented as mean ± SD; *p*-values were obtained from one-way analysis of variance (ANOVA); * indicates *p*-values adjusted for age, BMI and sex; *p* < 0.05 is considered significant. Superscripts A and B indicate the significance for control and prediabetes groups, respectively.

**Table 2:** Differences in Serum Cu, Fe and Zn according to T2DM status.

Trace Minerals	Non-T2DM	Prediabetes	T2DM	*p*-Value	*p*-Value *
Cu (mg/L)	2.7 ± 1.3	1.5 ± 0.5 ^A^	0.9 ± 0.6 ^A,B^	<0.001	<0.001
Fe (mg/L)	1.8 ± 0.4	1.8 ± 0.7	1.3 ± 0.5 ^A,B^	<0.001	<0.001
Zn (mg/L)	1.7 ± 0.4	2.1 ± 0.1 ^A^	1.7 ± 0.4 ^B^	<0.001	<0.001

Note: data presented as mean ± SD for normal variables but as median (first quartile–third quartile) for nonnormal variables; *p*-values were obtained from one-way analysis of variance (ANOVA); * indicates *p*-values adjusted for age, BMI and sex; *p* < 0.05 is considered significant. Superscripts A and B indicate significance for the control and prediabetes groups, respectively.

**Table 3:** Operating conditions of the instruments.

Radio Frequency Power	1600 W
Nebulizer gas flow	0.65 L/min
Lens Voltage	9.55 V
Analog Stage Voltage	−1745 V
Pulse Stage Voltage	950 V
Number of Replicates	3
Reading/Replicates	20
Scan Mode	Peak Hopping
Dwell Time	40 ms
Integration	1200 ms

## Data Availability

All data are contained within the article.
